# Enhancing Energy Storage Performance of 0.85Bi_0.5_Na_0.5_TiO_3_-0.15LaFeO_3_ Lead-Free Ferroelectric Ceramics via Buried Sintering

**DOI:** 10.3390/ma17164019

**Published:** 2024-08-13

**Authors:** Yixiao Zhang, Yuchen Jia, Jian Yang, Zixuan Feng, Shuohan Sun, Xiaolong Zhu, Haotian Wang, Shiguang Yan, Ming Zheng

**Affiliations:** 1 School of Materials Science and Physics, China University of Mining and Technology, Xuzhou 221116, China; ts22180124p31@cumt.edu.cn (Y.Z.);; 2 Key Laboratory of Inorganic Functional Materials and Devices, Shanghai Institute of Ceramics, Chinese Academy of Sciences, Shanghai 200050, China; 3 Key Laboratory of Polar Materials and Devices, Ministry of Education, East China Normal University, Shanghai 200241, China

**Keywords:** BNT-based ceramics, buried sintering, dielectric, ferroelectric, energy storage

## Abstract

Bismuth sodium titanate (Bi_0.5_Na_0.5_TiO_3_, BNT) ceramics are expected to replace traditional lead-based materials because of their excellent ferroelectric and piezoelectric characteristics, and they are widely used in the industrial, military, and medical fields. However, BNT ceramics have a low breakdown field strength, which leads to unsatisfactory energy storage performance. In this work, 0.85Bi_0.5_Na_0.5_TiO_3_-0.15LaFeO_3_ ceramics are prepared by the traditional high-temperature solid-phase reaction method, and their energy storage performance is greatly enhanced by improving the process of buried sintering. The results show that the buried sintering method can inhibit the formation of oxygen vacancy, reduce the volatilization of Bi_2_O_3_, and greatly improve the breakdown field strength of the ceramics so that the energy storage performance can be significantly enhanced. The breakdown field strength increases from 210 kV/cm to 310 kV/cm, and the energy storage density increases from 1.759 J/cm^3^ to 4.923 J/cm^3^. In addition, the energy storage density and energy storage efficiency of these ceramics have good frequency stability and temperature stability. In this study, the excellent energy storage performance of the ceramics prepared by the buried sintering method provides an effective idea for the design of lead-free ferroelectric ceramics with high energy storage performance and greatly expands its application field.

## 1. Introduction

Ferroelectric materials are widely used in the detection, conversion, processing, and storage of various kinds of information because of their excellent ferroelectric and piezoelectric properties [1,2,3,4,5]. However, traditional ferroelectric ceramics contain the Pb element, which will cause serious harm to the human body and the environment in the production, use, and waste of ceramics [6,7]. Among many lead-free ferroelectric materials, because Bi^2+^ and Pb^2+^ have a similar single-pair electron ^6^S_2_ structure, Bi_0.5_Na_0.5_TiO_3_ (BNT) ceramic has excellent ferroelectric properties [8]. BNT-based ceramics are considered to be one of the most likely to replace lead-free ferroelectric ceramics [9,10]. However, the low breakdown electric field strength and poor energy storage performance of pure BNT ceramic limit its application in electrical fields [11]. In order to improve the electrical properties of BNT ceramics, the modification of BNT-based ferroelectric ceramics has become the main research direction in the ferroelectric field. Some of these new BNT-based ferroelectric materials are already well known. For example, Jiang et al. [12] constructed 0.8Bi_0.5_Na_0.5_TiO_3_-0.2Ba_0.3_Sr_0.4_TiO_3_ and mixed 0.1NaNbO_3_ on this basis to obtain a good solid solution structure. A high energy storage density of 2.26 J/cm^3^ at 180 kV/cm was obtained, and the relaxation characteristics and dielectric temperature stability of the material were enhanced. Guo et al. [13] engineered and synthesized (1−*x*)(0.94Bi_0.5_Na_0.5_TiO_3_-0.06BaTiO_3_)-*x*BiMg_2/3_Nb_1/3_O_3_ solid solution to achieve the co-existence of tetragonal- and rhombohedral-phase PNRs in the perovskite structure, and an ultra-high energy density of 6.3 J/cm^3^ and an energy efficiency of 79.6% were obtained. Zhang et al. [14] found that the La element can improve the energy storage density and efficiency of 0.93 (Bi_0.5_Na_0.5_)TiO_3_-0.07Ba (Ti_0.945_Zr_0.055_)O_3_ ceramics. Gong et al. [15] found that Bi_0.9_La_0.1_FeO_3_ ceramics have a low leakage current density and high saturation polarization. At present, a large number of research works have made certain progress, such as controlling the sintering temperature [16,17], using a N_2_ atmosphere for sintering [18], adding the sintering aid CuO [19,20], and other methods. Improving the preparation process, such as the sintering process, is also expected to improve the performance of ceramics. Buried sintering refers to when the ceramic structure is buried under something (powder, etc.) for sintering [21,22]. For example, Fujii et al. [23] found that sintering BaTiO_3_-Bi(Mg_1/2_Ti_1/2_)O_3_-BiFeO_3_ ceramic samples in a bismuth-rich atmosphere, that is, sintering in a closed calcination powder crucible with the same composition, inhibited the volatilization of Bi_2_O_3_, thus enhancing the electrical properties of the ceramics. Li et al. [24] prepared Li_3+*x*_Mg_2_NbO_6_ ceramics with excellent microwave dielectric properties using the buried powder sintering method. The purpose of the buried sintering method is to prevent the material loss, volatilization, and combustion of the ceramic at a high temperature during sintering, resulting in the loss of bonding and disintegration of the billet and the inability to maintain its shape. Secondly, buried sintering can have the effect of isolating the air, which can prevent the reaction of the ceramic powder and the components in the air at high temperatures.

Therefore, in this study, 0.85Bi_0.5_Na_0.5_TiO_3_-0.15LaFeO_3_ (BNT-LFO) binary solid solution is constructed to study the relationship between its microstructure and electrical properties. More importantly, compared with ordinary sintering, the micro-structure of ceramics is optimized by using buried sintering, which greatly improves the energy storage properties of BNT-LFO ceramics. Besides doping modification, it provides a new design idea for improving the energy storage performance of BNT-based ceramics.

## 2. Materials and Methods

BNT-LFO ceramics are prepared by the high-temperature solid reaction method. The experimental raw materials are Bi_2_O_3_ (99%), Na_2_CO_3_ (99.9%), TiO_2_ (99%), La_2_O_3_ (99.99%), and Fe_2_O_3_ (99.9%). The experimental steps are as follows: According to the stoichiometric ratio, ball milling is carried out for 8 h. After drying, the pre-burning temperature is 650 °C, heat preservation is carried out for 4 h, and the heating rate is 3 °C/min. Next, 5%PVA is used for granulation, and then tablet pressing is carried out (applied pressure: 0.6 Pa, ceramic area: 12.56 mm^2^, and thickness: 0.3 mm). The heat is kept at 650 °C for 3 h to discharge the glue (PVA), and then it is raised to 1100 °C for 2 h for sintering, and the heating rate is 3 °C/min, and then it is naturally cooled to room temperature. The powder used for buried sintering is made of pre-fired powder. The crystal structures of the ceramic samples are analyzed by X-ray diffractometry (XRD, D8 ADVANCE) with Cu *K_α1_* radiation (λ = 1.5406 Å). The surface morphology of the samples is characterized by scanning electron microscopy (SEM, JEOL JEM-7800F, Akishima, Japan). The dielectric test system (Wayne Kerr 6500B, Shenzhen, China) is used to test the dielectric properties of ceramics at room temperature. The ferroelectric and energy storage properties of ceramics are tested by a ferroelectric analysis facility (TF-3000, aixACCT, Aachen, Germany) at room temperature, and variable temperature energy storage is carried out at 20–100 °C.

## 3. Results and Discussion

**Figure 1:**
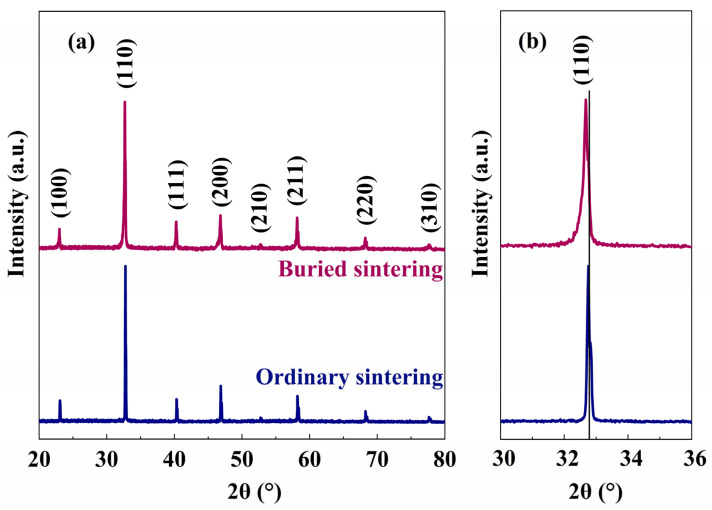
The XRD patterns of the BNT-LFO ceramics under ordinary sintering and buried sintering in the 2θ ranges of (**a**) 20–80° and (**b**) 30–36°.

The XRD patterns of BNT-LFO ceramics under ordinary sintering and buried sintering are shown in Figure 1a. All ceramics have a pure perovskite structure without introducing the second phase, indicating that LFO has completely entered into the BNT lattice, forming a good solid solution. The main diffraction peak (110) is amplified, as shown in Figure 1b, and it is found that the diffraction peak position of the buried sintered ceramic is shifted to a lower angle compared with that of the ordinary sintered ceramic. According to the Bragg equation, the spacing between the crystal faces increases. Buried sintering can reduce the evaporation of Bi_2_O_3_ and the generation of oxygen vacancy, and the smaller the vacancy, the larger the crystal plane spacing. Therefore, the distance between the ceramic crystal faces after buried sintering is larger, and the position of the main diffraction peak is slightly shifted to the left side. In addition, the bottom of the XRD diffraction peak of the buried sintered ceramic shows a wider peak, which is caused by the reduction in the grain size of the ceramic after buried sintering.

**Figure 2:**
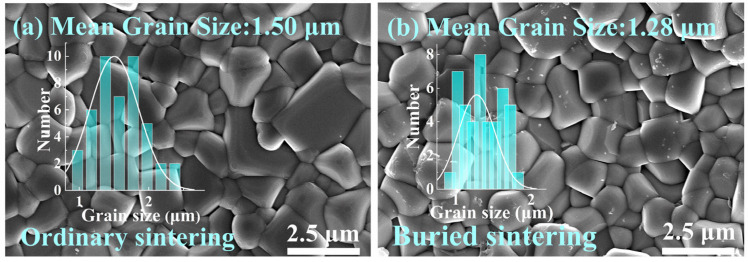
SEM pictures of (**a**) ordinary sintered BNT-LFO ceramic and (**b**) buried sintered BNT-LFO ceramic; the insets are the distributions of the grain size.

The surface microscopic morphologies of the ordinary sintered and buried sintered BNT-LFO ceramics are shown in Figure 2. The insets are the grain distribution data obtained by using the Nano Measurer (2009) software and origin (2018) software. The mean grain size of the buried sintered ceramic is 1.28 μm, which is significantly lower than that of the ordinary sintered ceramic (1.50 μm), showing a reduction of 14.7%. In addition, the grain size distribution of the ceramics prepared by the buried sintering method is more concentrated, the grain size is more uniform, and the number of abnormally coarse grains is significantly reduced, which is expected to improve the electrical properties of BNT-LFO ferroelectric ceramics.

**Figure 3:**
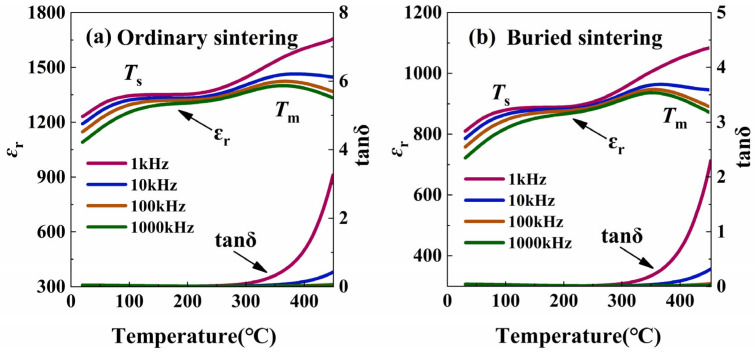
Dielectric temperature spectra of (**a**) ordinary sintered BNT-LFO ceramic and (**b**) buried sintered BNT-LFO ceramic.

The dielectric constant (*ε*_r_) and dielectric loss (tanδ) curves of the ordinary sintered and buried sintered BNT-BFO ceramics are shown in Figure 3. The measurement frequencies are 1 kHz, 10 kHz, 100 kHz, and 1 MHz. Two dielectric anomaly peaks can be found, where *T*_s_ occurs at lower temperatures (~100 °C), which is believed to be characteristic of relaxed ferroelectrics and is associated with thermal relaxation in the PNRs. *T*_m_ occurs at higher temperatures (~380 °C), which corresponds to the transition from the rhombohedral phase to the tetragonal phase [25,26,27]. The ε_r_ of the buried sintered ceramic decreased, mainly because this method inhibits the volatilization of Bi_2_O_3_, which causes charge fluctuation. After the ceramic undergoes buried sintering, the tanδ is reduced, which can effectively reduce the energy loss, providing the basis for obtaining excellent electrical properties.

**Figure 4:**
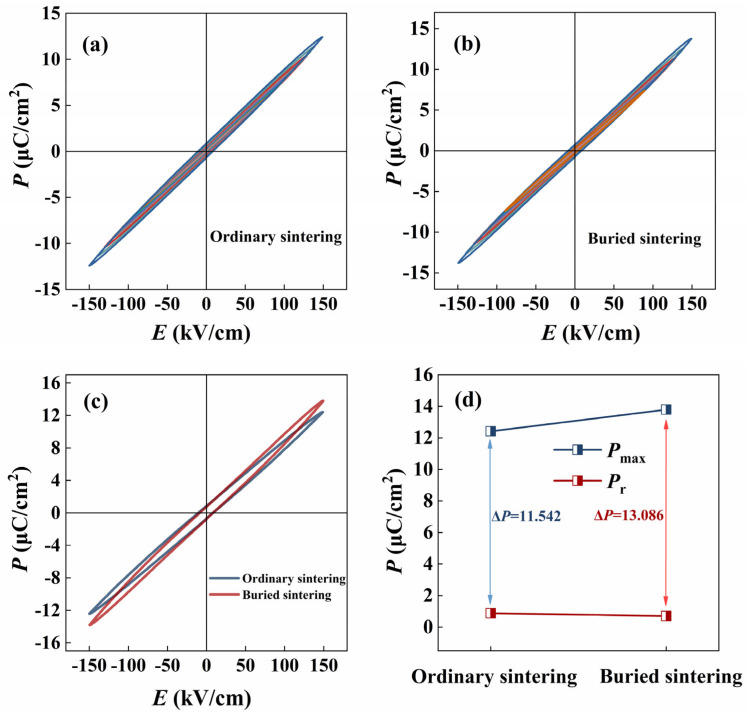
The bipolar *P*-*E* loops of (**a**) ordinary sintered and (**b**) buried sintered BNT-LFO ceramics. (**c**) The bipolar *P*-*E* loops of ordinary sintered and buried sintered BNT-LFO ceramics at 150 kV/cm. (**d**) The variation in the *P*_max_ and *P*_r_ values of ordinary sintered and buried sintered BNT-LFO ceramics at 150 kV/cm. The curves of different colors represent the bipolar *P*-*E* loops under different electric fields. Due to the different materials a and b, the bipolar *P*-*E* loops are different.

At room temperature, the bipolar *P*-*E* loops of the ceramics under different electric fields are shown in Figure 4a,b. The bipolar *P*-*E* loop of the ceramics under a 150 KV/cm electric field is summarized in Figure 4c. The ferroelectric property of the buried sintered ceramic is obviously better than that of the ordinary sintered one. The key parameters, maximum polarization (*P*_max_), remanent polarization (*P*_r_), and Δ*P* (*P*_max_ − *P*_r_) are summarized, as shown in Figure 4d. By comparison, it is found that the *P*_max_ value increased from 12.422 µC/cm^2^ to 13.793 µC/cm^2^, and the *P*_r_ value decreased from 0.880 µC/cm^2^ to 0.708 µC/cm^2^ for the buried sintered BNT-LFO ceramics. Δ*P* increased from 11.542 µC/cm^2^ to 13.086 µC/cm^2^. The increase in Δ*P* is expected to improve the energy storage performance of the ceramics.

**Figure 5:**
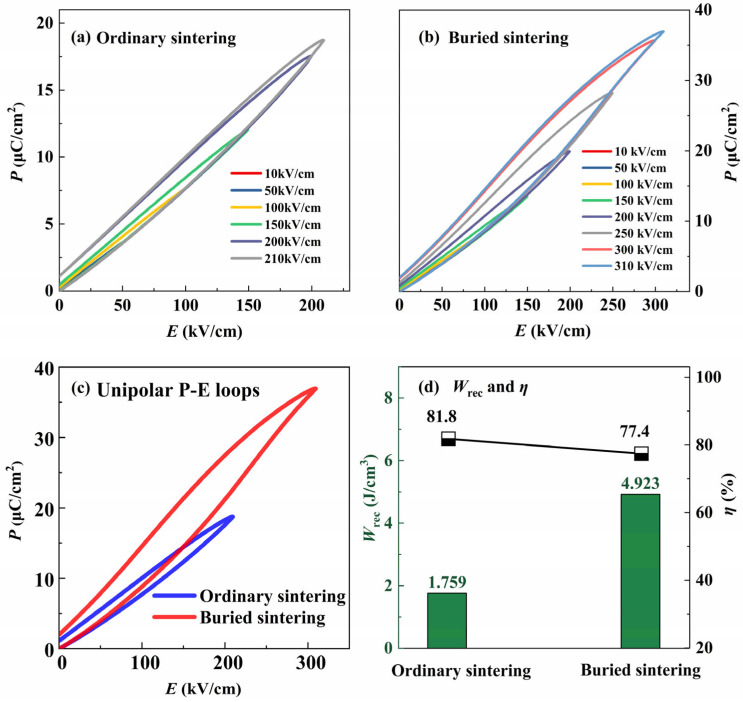
The unipolar *P*-*E* loops of (**a**) ordinary sintered and (**b**) buried sintered BNT-LFO ceramics. (**c**) The unipolar *P*-*E* loops of ordinary sintered and buried sintered BNT-LFO ceramics at their maximum electric fields. (**d**) The variation in the *W*_rec_ and *η* values of ordinary sintered and buried sintered BNT-LFO ceramics.

In order to explore the influence of the buried sintering method on the energy storage performance of BNT-LFO ceramics, we tested the unipolar *P*-*E* loops of ordinary sintered and buried sintered ceramics, and the test frequency was 10 Hz. The test results are shown in Figure 5a,b, respectively. The breakdown electric field strength of the buried sintered ceramic is up to 310 kV/cm, which is 1.48 times as large as that of the ordinary sintered ceramic (210 kV/cm). The unipolar *P*-*E* loops of the ordinary sintered and buried sintered ceramics under their maximum electric field is shown in Figure 5c. *P*_max_ increases from 18.741 µC/cm^2^ (ordinary sintering) to 36.968 µC/cm^2^ (buried sintering), which is an increase of 97.3%. According to the unipolar *P*-*E* loops, the discharge energy storage density (*W*_rec_) and energy storage efficiency (η) are calculated. The total energy storage density (*W*), *W*_rec_, and *η* of the ferroelectric materials are calculated as follows [28]:

(1) $$W=\int_{0}^{P_{max}}EdP$$

(2) $$W_{\mathrm{rec}}\;\int_{P_{r}}^{P_{max}}EdP$$

*η* = (*W*_rec_/*W*) × 100% (3)

The *W*_rec_ and *η* values of the ordinary sintered and buried sintered ceramics are shown in Figure 5d. After buried sintering, the *W*_rec_ of the BNT-LFO ceramics can be increased from 1.759 J/cm^3^ to 4.923 J/cm^3^, which is a huge increase of 179.9%. The *η* decreased slightly from 81.8% to 77.4%, which is a decrease of about 5.7%. The microstructure of the buried sintered BNT-LFO ceramic is more uniform and compact, and the average grain size decreases, so it shows a larger breakdown electric field strength than ordinary ceramic. The *W*_rec_ and *η* values of the ceramics are improved. This result shows that BNT-LFO ceramics can be changed by buried sintering to obtain better energy storage performance, which greatly expands its application in the field of electricity.

**Figure 6:**
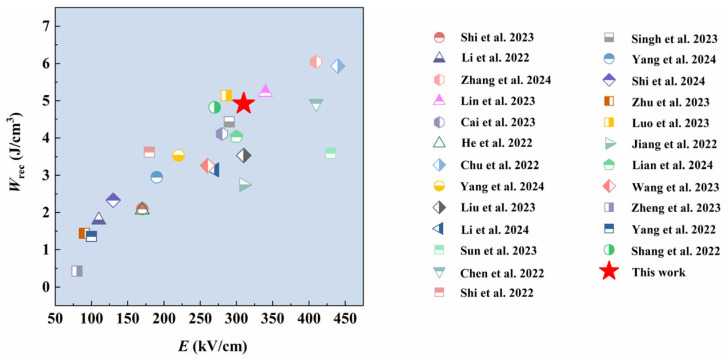
A comparison of the *W*_rec_ in this work with that of other previously published lead-free BNT-based ceramics [29,30,31,32,33,34,35,36,37,38,39,40,41,42,43,44,45,46,47,48,49,50,51,52].

The *W*_rec_ of buried sintered BNT-LFO ceramic is compared with other previously published lead-free BNT-based ceramics, as shown in Figure 6 [29,30,31,32,33,34,35,36,37,38,39,40,41,42,43,44,45,46,47,48,49,50,51,52]. The energy storage ability is compared between this work and other previously published lead-free BNT-based ceramics, and the results are shown in Table 1. The breakdown electric field strength of ceramic can reach 310 kV/cm, and the *W*_rec_ is as high as 4.923 J/cm^3^. The results show that the buried sintering progress is a good strategy for improving the energy storage performance of lead-free ferroelectric ceramics.

**Figure 7:**
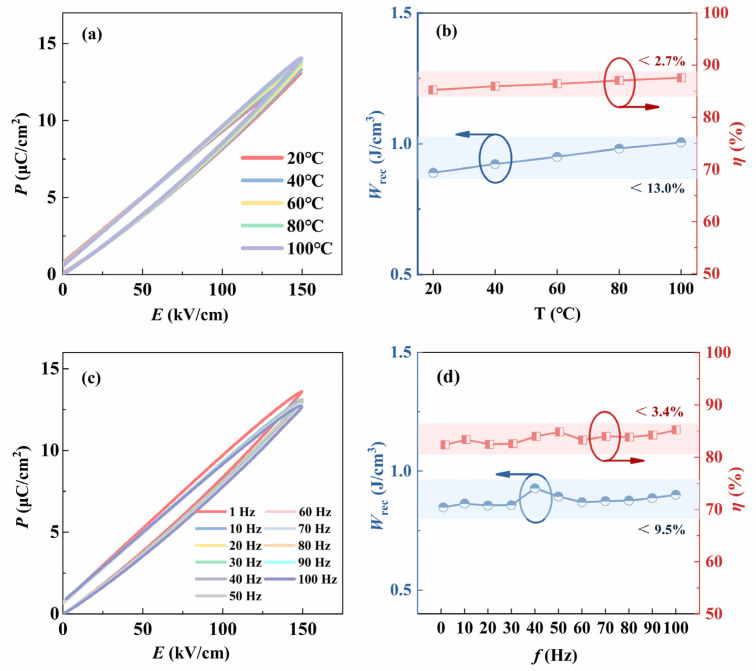
(**a**) The unipolar *P*-*E* loops of buried sintered BNT-LFO ceramic under different temperatures. (**b**) The variation in *W*_rec_ and *η* of buried sintered BNT-LFO ceramic under different temperatures. (**c**) The unipolar *P*-*E* loops of buried sintered BNT-LFO ceramic under different frequencies. (**d**) The variation in *W*_rec_ and *η* of buried sintered BNT-LFO ceramic under different frequencies.

Excellent temperature stability and frequency stability are the prerequisites for the material to ensure the stable operation of the device in practical applications. Therefore, we test the energy storage temperature stability and frequency stability of the buried sintered BNT-LFO ceramic. Figure 7a shows the unipolar *P*-*E* loops of the ceramic at different temperatures. The change curves of *W*_rec_ and *η* under the electric field of 150 kV/cm with the increase in temperature are shown in Figure 7b. In the temperature range of 30 °C to 100 °C, the fluctuation in *W*_rec_ is less than 2.7%, and the fluctuation in *η* is less than 13.0%. Figure 7c shows the unipolar *P*-*E* loops of the ceramic at different frequencies. The change curves of *W*_rec_ and *η* under the electric field of 150 kV/cm with the increase in frequency are shown in Figure 7d. The fluctuations in *W*_rec_ and *η* are less than 3.5% and 9.5%, respectively. The results show that the ceramics have excellent energy storage temperature stability and frequency stability.

## 4. Conclusions

In summary, BNT-LFO ceramics are prepared by the traditional high-temperature solid state reaction method, and the ceramics are made into porcelain by ordinary sintering and buried sintering. The microstructure and dielectric, ferroelectric, and energy storage properties of the ceramics are studied. The results show that buried sintered ceramic can inhibit the volatilization of Bi_2_O_3_, reduce the generation of oxygen vacancy, make the microstructure more uniform, and reduce the main grain size. The breakdown electric field strength increases from 210 kV/cm to 310 kV/cm. *W*_rec_ increases from 1.759 J/cm^3^ to 4.923 J/cm^3^, which is an increase of 179.9%. It shows good stability in the energy storage temperature and frequency. In this study, the advantages of buried sintered ceramic from the microscopic level to electrical properties are deeply discussed, which provides a new method for further improving the energy storage performance of BNT-based lead-free ferroelectric ceramics and lays a theoretical foundation for the development of BNT-based lead-free ferroelectric ceramics with high storage performance.

## Figures and Tables

**Table 1:** A comparison of the energy storage ability in this work and other previously published lead-free BNT-based ceramics.

Composition	*E* (kV/cm)	*W*_rec_ (J/cm^3^)	*η* (%)	Ref.
(Ba_0.3_Sr_0.7_)_0.5_(Bi_0.5_Na_0.5_)_0.5_TiO_3_ + 14 wt% GS	170	2.1	65.2	[29]
Ba_0.105_Na_0.325_Sr_0.185_Bi_0.385_TiO_3_	110	1.8	~73	[30]
0.45(Bi_0.5_Na_0.5_)TiO_3_-0.55(Sr_0.7_Bi_0.2_)TiO_3_-0.3(Bi_0.2_Na_0.2_Ba_0.2_Sr_0.2_Ca_0.2_)(Ti_0.9_Nb_0.1_)O_3_	410	6.04	85	[31]
Bi_0.5_N_0.5_TiO_3_-SrTiO_3_-0.04Sr_2_NaNb_5_O_15_	340	5.22	93.87	[32]
(65%(0.92Bi_0.5_Na_0.5_TiO_3_-0.08Bi(Mg_0.3_Zr_0.6_)O_3_)-35%(0.6BaTiO_3_-0.4NaNbO_3_)	280	4.11	95.6	[33]
0.54Bi_0.5_Na_0.5_TiO_3_-0.06BaTiO_3_-0.4Bi_0.2_Sr_0.7_Ti_0.96875_Nb_0.125_O_3_	170	2.07	94.5	[34]
[(Bi_0.5_Na_0.5_)_0.94_Ba_0.06_]_0.82_La_0.12_TiO_3_	440	5.93	77.6	[35]
0.7Bi_0.5_Na_0.5_TiO_3_-0.2BaZr_0.3_Ti_0.7_O_3_-0.1NaNbO_3_	220	3.53	93.5	[36]
0.85(0.8525BNT–0.10995BKT–0.03755BT)0.15Sr(Mg_1/3_Nb_2/3_)O_3_	310	3.53	86.3	[37]
0.6Bi_0.5_Na_0.5_TiO_3_-0.4SrTiO_3_(@Si-TSS)	270	3.14	86.21	[38]
0.9[0.88Ba_0.6_Ca_0.4_TiO_3_-0.12Bi(Mg_2/3_(Nb_0.85_Ta_0.15_)_1/3_)O_3_]-0.1Bi_0.5_Na_0.5_TiO_3_	430	3.59	90.86	[39]
0.7Na_0.5_Bi_0.5_TiO_3_-0.3NaNbO_3_/7 wt%CaZr_0.5_Ti_0.5_O_3_	410	4.93	93.3	[40]
0.92Bi_0.5_Na_0.5_TiO_3_–0.08LiNbO_3_	180	3.62	80.8	[41]
0.65Bi_0.5_Na_0.4_K_0.1_TiO_3_-0.35[2/3SrTiO_3_-1/3Bi(Mg_2/3_Nb_1/3_)O_3_]	290	4.43	86	[42]
0.9Bi_0.5_Na_0.5_TiO_3_−0.1BaZr_0.3_Ti_0.7_O_3_:0.6mol%Er^3+^	190	2.95	51.3	[43]
Ba_0.087_Sr_0.176_Bi_0.385_Na_0.325_TiO_3_	130	2.33	64.5	[44]
(Bi_0.5_Na_0.5_)_0.65_(Ba_0.3_Sr_0.7_)_0.35_(Ti_0.98_Ce_0.02_)O_3_+2wt%Nb_2_O_5_	90	1.44	84.1	[45]
0.5Bi_0.5_Na_0.5_TiO_3_-0.5NaNbO_3_	286	5.14	79.65	[46]
0.75Bi_0.5_Na_0.5_TiO_3_-0.25CaTiO_3_	310	2.74	91	[47]
0.7[0.85(0.84Bi_0.5_Na_0.5_TiO_3_-0.16Bi0.5K_0.5_TiO_3_)-0.15BiMg_2/3_Nb_1/3_O_3_]-0.3Sr_0.7_La_0.2_TiO_3_	300	4.03	85.2	[48]
0.53(Bi_0.5_Na_0.5_)TiO_3_-0.07BaTiO_3_-0.4(Sr_0.7_Bi_0.2_)TiO_3_	260	3.26	90.3	[49]
0.94Bi_0.5_Na_0.5_TiO_3_-0.06BaTiO_3_:1mol%Er^3+^	80	0.429	~48	[50]
[0.93(Bi_0.51_Na_0.5_)0.07Ba)]Ti_0.9925_(Sr_1/3_Nb_2/3_)_0.0075_O_3_	100	1.36	~61	[51]
0.85(0.75Bi_0.5_Na_0.4_K_0.1_TiO_3_-0.25SrTiO_3_)-0.15Bi(Mg_0.5_Ti_0.5_)O_3_	270	4.82	84.9	[52]
0.85Bi_0.5_Na_0.5_TiO_3_-0.15LaFeO_3_ (buried sintering)	310	4.923	77.4	This work

## Data Availability

The original contributions presented in the study are included in the article, further inquiries can be directed to the corresponding author.

## References

[1] Zhu Z.Y.S., Persson A.E.O., Wernersson L.E. (2023). Reconfigurable signal modulation in a ferroelectric tunnel field-effect transistor. Nat. Commun..

[2] He T.F., Cao Z.Z., Li G.R., Jia Y.M., Peng B.L. (2022). High efficiently harvesting visible light and vibration energy in (1−*x*)AgNbO_3_−*x*LiTaO_3_ solid solution around antiferroelectric-ferroelectric phase boundary for dye degradation. J. Adv. Ceram..

[3] Xue F., He X., Ma Y.C., Zheng D.X., Zhang C.H., Li L.J., He J.H., Yu B., Zhang X.X. (2021). Unraveling the origin of ferroelectric resistance switching through the interfacial engineering of layered ferroelectric-metal junctions. Nat. Commun..

[4] Jin T.Y., Mao J.Y., Gao J., Han C., Wee A.T.S., Loh K.P., Chen W. (2022). Ferroelectrics-integrated two-dimensional devices toward next-generation electronics. ACS Nano.

[5] Niu X., Jian X.D., Gong W.P., Zhang G.Z., Jiang S.L., Yu K., Zhao X.B., Yao Y.B., Tao T., Liang B. (2022). Field-driven merging of polarizations and enhanced electrocaloric effect in BaTiO_3_-based lead-free ceramics. J. Adv. Ceram..

[6] Zhong X.C., Lin Z.C., Chen C., Wang R.X., Zhong S.P., Xu Z.F. (2021). The role of ZnFe_2_O_4_ in the electrochemical performance of Pb-ceramic composite anode in sulfuric acid solution. Hydrometallurgy.

[7] Chen Y., Li L.F., Zhou Z., Wang Y.Y., Chen Q., Wang Q.Y. (2023). La_2_O_3_-modified BiYbO_3_-Pb(Zr,Ti)O_3_ termary piezoelectric ceramics with enhanced electrical properties and thermal depolarization temperature. J. Adv. Ceram..

[8] Kounga A.B., Zhang S.T., Jo W., Granzow T., Rödel J. (2008). Morphotropic phase boundary in (1 − *x*)Bi_0.5_Na_0.5_TiO_3−_*x*K_0.5_Na_0.5_NbO_3_ lead-free piezoceramics. Appl. Phys. Lett..

[9] Zidani J., Alaoui I.H., Zannen M., Birks E., Chchiyai Z., Majdoub M., Manoun B., Marssi M.E., Lahmar A. (2024). On the lanthanide effect on functional properties of 0.94Na_0.5_Bi_0.5_TiO_3_-0.06BaTiO_3_ ceramic. Materials.

[10] Uddin S., Ahmad A., Nasir M.F., Zaman A., Algahtani A., Tirth V., Zheng G.P. (2024). Effect of BiFeO_3_ on the ferroelectric and energy storage properties of (Bi_1/2_Na_1/2_)_0.94_Ba_0.06_TiO_3_ based compositions. Inorg. Chem. Commun..

[11] Pan Y., Dai Z.H., Liu C.X., Zhao X., Yasui S., Cong Y., Gu S.T. (2024). High energy storage properties of Nd(Mg_2/3_Nb_1/3_)O_3_ modified Bi_0.5_Na_0.5_TiO_3_ lead-free ceramics. J. Mater. Sci..

[12] Jiang Z.H., Yang Z.Y., Yuan Y., Tang B., Zhang S.R. (2021). High energy storage properties and dielectric temperature stability of (1 − *x*)(0.8Bi_0.5_Na_0.5_TiO_3_-0.2Ba_0.3_Sr_0.4_TiO_3_)-*x*NaNbO_3_ lead-free ceramics. J. Alloys Compd..

[13] Guo B., Yan Y., Tang M.Y., Wang Z.Y., Li Y., Zhang L.Y., Zhang H.B., Jin L., Liu G. (2021). Energy storage performance of Na_0.5_Bi_0.5_TiO_3_ based lead-free ferroelectric ceramics prepared via non-uniform phase structure modification and rolling process. Chem. Eng. J..

[14] Zhang X.R., Xiao Y.A., Du B.N., Li Y.M., Wu Y.D., Sheng L.Y., Tan W.C. (2021). Improved non-piezoelectric electric properties based on La modulated ferroelectric-ergodic relaxor transition in (Bi_0.5_Na_0.5_)TiO_3_-Ba(Ti, Zr)O_3_ ceramics. Materials.

[15] Gong Y.F., Wu P., Liu W.F., Wang S.Y., Liu G.Y., Rao G.H. (2012). Switchable ferroelectric diode effect and piezoelectric properties of Bi_0.9_La_0.1_FeO_3_ Ceramics. Chin. Phys. Lett..

[16] Kwon Y.H., Lee G.H., Koh J.H. (2015). Effects of sintering temperature on the piezoelectric properties of (Bi,Na)TiO_3_-based composites for energy harvesting applications. Ceram. Int..

[17] Qiu Y.Z., Yu Z.D. (2023). Effect of sintering temperature on structure and electrical properties of ZnO-added (Bi_0.5_Na_0.5_)_0.94_Ba_0.06_TiO_3_ lead-free ceramics. J. Mater. Sci.-Mater. Electron..

[18] Leng S.L., Jia F.H., Zhong Z.K., Yang Q.F., Li G.R., Zheng L.Y. (2015). Fabrication of High *T*_c_ BaTiO_3_-(Bi_0.5_Na_0.5_)TiO_3_ Lead-free positive temperature coefficient of resistivity ceramics. J. Inorg. Mater..

[19] Ahn C.W., Kim H.S., Woo W.S., Won S.S., Seog H.J., Chae S.A., Park B.C., Jang K.B., Ok Y.P., Chong H.H. (2015). Low-temperature sintering of Bi_0.5_(Na,K)_0.5_TiO_3_ for multilayer ceramic actuators. J. Am. Ceram. Soc..

[20] Tian H.Y., Kwok K.W., Chan H.L.W., Buckley C.E. (2007). The effects of CuO-doping on dielectric and piezoelectric properties of Bi_0.5_Na_0.5_TiO_3_-Ba(Zr,Ti)O_3_ lead-free ceramics. J. Mater. Sci..

[21] Kong D.K., Guo A.F., Hu Y.B., Zhou X.Y., Wu H.L., Li X.J., Qu P., Wang S.Q., Guo S. (2023). Alumina-based ceramic cores prepared by vat photopolymerization and buried combustion method. Mater. Today Commun..

[22] Chen R.Y., Xie K.S., Zhu H.P., He Q., Li S.S., Wen H.M. (2023). Improving strength and microstructure of SiC reticulated porous ceramic through in-situ generation of SiC whiskers within hollow voids. Ceram. Int..

[23] Fujii I., Mitsui R., Nakashima K., Kumada N., Yabuta H., Shimada M., Watanabe T., Miura K., Wada S. (2013). Effect of sintering condition and V-doping on the piezoelectric properties of BaTiO_3_-Bi(Mg_1/2_Ti_1/2_)O_3_-BiFeO_3_ ceramics. J. Cearm. Soc. Jpn..

[24] Li H., Liu Y.S., Liu Y.S., Zeng Q.F., Hu K.H., Lu Z.G., Liang J.J. (2020). Effect of burying sintering on the properties of ceramic cores via 3D printing. J. Manuf. Process..

[25] Long C., Su Z., Song H., Xu A., Liu L., Li Y., Zheng K., Ren W., Wu H., Ding X. (2024). Excellent energy storage properties with ultrahigh *W*_rec_ in lead-free relaxor ferroelectrics of ternary Bi_0.5_Na_0.5_TiO_3_-SrTiO_3_-Bi_0.5_Li_0.5_TiO_3_ via multiple synergistic optimization. Energy Storage Mater..

[26] Fan J.T., He G., Cao Z.Z., Cao Y.F., Long Z., Hu Z.G. (2023). Ultrahigh energy-storage density of a lead-free 0.85Bi_0.5_Na_0.5_TiO_3_-0.15Ca(Nb_0.5_Al_0.5_)O_3_ ceramic under low electric fields. Inorg. Chem. Front..

[27] Che Z.Y., Ma L., Luo G.G., Xu C., Cen Z.Y., Feng Q., Chen X.Y., Ren K.L., Luo N.N. (2022). Phase structure and defect engineering in (Bi_0.5_Na_0.5_)TiO_3_-based relaxor antiferroelectrics toward excellent energy storage performance. Nano Energy.

[28] Guan P.F., Zhang Y.X., Yang J., Zheng M. (2023). Effect of Sm^3+^ doping on ferroelectric, energy storage and photoluminescence properties of BaTiO_3_ ceramics. Ceram. Int..

[29] Shi X.H., Li K., Shen Z.Y., Liu J.Q., Chen C.Q., Zeng X.J., Zhang B., Song F.S., Luo W.Q., Wang Z.M. (2023). BS_0.5_BNT-based relaxor ferroelectric ceramic/glass-ceramic composites for energy storage. J. Adv. Ceram..

[30] Li Z.P., Li D.X., Shen Z.Y., Zeng X.J., Song F.S., Luo W.Q., Wang X.C., Wang Z.M., Li Y.M. (2022). Remarkably enhanced dielectric stability and energy storage properties in BNT-BST relaxor ceramics by A-site defect engineering for pulsed power applications. J. Adv. Ceram..

[31] Zhang X., Zhang F., Niu Y.W., Zhang Z.Q., Lei X.Q., Wang Z.J. (2024). Excellent energy storage performance of perovskite high-entropy Oxide-modified (Bi_0.5_Na_0.5_)TiO_3_-based ceramics. ACS Appl. Electron. Mater..

[32] Lin Y.Z., Wan R.F., Zheng P., Li Z.H., Wang Y.K., Fan Q.L., Zheng L., Zhang Y., Bai W.F. (2023). Achieving remarkable energy storage performances under low electric field in Bi_0.5_N_0.5_TiO_3_-SrTiO_3_-based relaxor ferroelectric ceramics via a heterostructure doping strategy. ACS Appl. Electron. Mater..

[33] Cai Z.M., Yang H., Zhu C.Q., Li S.H., Luo B.C., Li A.Y., Li X.H., Tian Z.B., Feng P.Z. (2023). Local heterogeneous polarization enhanced superior low-field energy storage performance in lead-free relaxor ferroelectric ceramics. ACS Sustain. Chem. Eng..

[34] He J., Liu X., Zhao Y., Du H., Zhang T., Shi J. (2022). Dielectric stability and energy-storage performance of BNT-based relaxor ferroelectrics through Nb^5+^ and its excess modification. ACS Appl. Electron. Mater..

[35] Chu B.K., Hao J.G., Li P., Li Y.C., Li W., Zheng L.M., Zeng H.R. (2022). High-energy storage properties over a broad temperature range in La-modified BNT-based lead-free ceramics. ACS Appl. Mater. Interfaces.

[36] Yang J., Zhu X.L., Wang H.T., Zhang Y.X., Guan P.F., Yan S.G., Zheng M. (2024). Achieving outstanding energy storage behaviors via combinatorial optimization design in BNT-based relaxor ferroelectric ceramics under medium-low electric fields. J. Mater. Chem. C.

[37] Liu T.Y., Yan B., Ma J.X., He Q., An L.N., Chen K.P. (2023). Enhanced energy storage properties in BNT-based ceramics with a morphotropic phase boundary modified by Sr(Mg_1/3_Nb_2/3_)O_3_. J. Mater. Chem. C.

[38] Li X.H., Zhu C.Q., Li S.H., Li A.Y., Liang L.Q., Cai Z.M., Feng P.Z. (2024). Enhancing energy storage density of BNT-ST-based ceramics by a stepwise optimization strategy on the breakdown strength. J. Eur. Ceram. Soc..

[39] Sun M.Z., Wang X.M., Li P., Du J., Fu P., Hao J.G., Li W., Zhai J.W. (2023). Realizing ultrahigh breakdown strength and ultrafast discharge speed in novel barium titanate-based ceramics through multicomponent compounding strategy. J. Eur. Ceram. Soc..

[40] Chen Y., Huang Y., Zuo Y.D., Wang H.S., Liu K., Fan B.Y., Zhang Q.F., Zhang G.Z., Jiang S.L., Shen M. (2022). Enhanced energy storage property achieved in Na_0.5_Bi_0.5_TiO_3_-based ferroelectric ceramics via composition design and grain size tuning. J. Eur. Ceram. Soc..

[41] Shi W.J., Zhang L.Y., Jing R.Y., Hu Q.Y., Zeng X.Y., Alikin D.O., Shur V.Y., Wei X.Y., Gao J.H., Liu G. (2022). Relaxor antiferroelectric-like characteristic boosting enhanced energy storage performance in eco-friendly (Bi_0.5_Na_0.5_)TiO_3_-based ceramics. J. Eur. Ceram. Soc..

[42] Singh A., Kharangarh P., Gupta V. (2023). Enhanced energy storage efficiency with superior thermal stability under low electric field and large electric field driven strain in environment- friendly Bi_0.5_Na_0.5_TiO_3_ based ferroelectric modified with LiNbO_3_. J. Alloys Compd..

[43] Yang J., Guan P.F., Zhang Y.X., Zhu X.L., Wang H.T., Yang C., Zheng M. (2024). High energy storage density achieved in BNT-based ferroelectric translucent ceramics under low electric fields. J. Am. Ceram. Soc..

[44] Shi X.H., Li Z.P., Shen Z.Y., Song F.S., Luo W.Q., Zeng X.J., Wang Z.M., Li Y.M. (2024). Ba^2+^/Sr^2+^ regulation in A-site vacancy-engineered B_0.015+1.5*x*_S_0.245-1.5*x*0.03_BNT relaxor ceramics for energy storage. J. Am. Ceram. Soc..

[45] Zhu W., Guo H.H., Shen Z.Y., Song F.S., Luo W.Q., Wang Z.M., Li Y.M. (2023). Boosting dielectric temperature stability in BNBST-based energy storage ceramics by Nb_2_O_5_ modification. J. Am. Ceram. Soc..

[46] Luo W.X., Wu M.X., Han Y.F., Zhou X., Liu L.J., He Q.W., Ren P.R., Yang H.M., Yang H., Wang Q. (2023). Enhanced optical transmittance and energy-storage performance in NaNbO_3_-modified Bi_0.5_Na_0.5_TiO_3_ ceramics. J. Am. Ceram. Soc..

[47] Jiang Z.H., Yuan Y., Yang H.C., Li E.Z., Zhang S.R. (2022). Excellent thermal stability and energy storage properties of lead-free Bi_0.5_Na_0.5_TiO_3_-based ceramic. J. Am. Ceram. Soc..

[48] Lian H.L., Liang X.J., Shi M., Liu L.N., Chen X.M. (2024). Improved dielectric temperature stability and energy storage properties of BNT-BKT-based lead-free ceramics. Ceram. Int..

[49] Wang D., Chu B.K., Li P., Han W.F., Kong Y.X., Fu P., Li Y.C., Hao J.G., Li W. (2023). Improving the energy storage performance of (Bi_0.5_Na_0.5_)TiO_3_-BaTiO_3_ based ceramics via (Sr_0.7_Bi_0.2_)TiO_3_ modification. Ceram. Int..

[50] Zheng M., Guan P.F., Yang J., Zhang Y.X. (2023). Microstructure and composition driven ferroelectric properties of Er^3+^ doped lead-free multifunctional 0.94Bi_0.5_Na_0.5_TiO_3_-0.06BaTiO_3_ ceramics. Ceram. Int..

[51] Yang Y., Jing R., Wang J., Lu X., Du H., Jin L. (2022). Large electrostrain and high energy-storage properties of (Sr_1/3_Nb_2/3_)^4+^-substituted (Bi_0.51_Na_0.5_)TiO_3_-0.07BaTiO_3_ lead-free ceramics. Ceram. Int..

[52] Shang K.L., Shi W.J., Yang Y.L., Zhang L.Y., Hu Q.Y., Wei X.Y., Jin L. (2022). Medium electric field-induced ultrahigh polarization response and boosted energy-storage characteristics in BNT-based relaxor ferroelectric polycrystalline ceramics. Ceram. Int..

